# *Osteopathic Medicine and Primary Care: *one journal, two audiences

**DOI:** 10.1186/1750-4732-4-1

**Published:** 2010-01-12

**Authors:** John C Licciardone

**Affiliations:** 1 The Osteopathic Research Center, University of North Texas Health Science Center, Fort Worth, TX, USA

## Abstract

*Osteopathic Medicine and Primary Care *(OMPC) enters its fourth year of operation in 2010 under the umbrella of BioMed Central. *Osteopathic Medicine and Primary Care *strives to promote and advance research and scholarly work within the fields of osteopathic medicine and primary care. In so doing, OMPC welcomes submissions from clinicians within both the osteopathic and allopathic medical professions, and from other professionals having interests in primary care, including health care delivery, public health, and evidence-based medicine. *Osteopathic Medicine and Primary Care *offers fair and expeditious peer review (mean time from submission to publication, 118 days), retention of copyright for authors, unlimited online distribution and access without charge to readers, indexing in PubMed, and archiving in PubMed Central. In 2010, there will be an increased availability of waivers or discounts of article processing charges via several mechanisms for eligible authors who submit qualified manuscripts, especially in the field of primary care.

## An historical perspective of *Osteopathic Medicine and Primary Care*

*Osteopathic Medicine and Primary Care *(OMPC) was established to provide the osteopathic research and academic communities with a much-needed mechanism for efficiently publishing and widely disseminating scholarly works. Since its appearance in January 2007, OMPC has fulfilled that need and maintained its standing within the osteopathic profession by being the only osteopathic journal to consistently offer fair and expeditious peer review, retention of copyright for authors, unlimited online distribution and access without charge to readers, indexing in PubMed, and archiving in PubMed Central. *Osteopathic Medicine and Primary Care *is published as an independent journal under the umbrella of BioMed Central (http://www.biomedcentral.com), the leading global open access publisher. *Osteopathic Medicine and Primary Care *publishes scholarly works consisting of original research, reviews, study protocols, short reports, commentaries, and editorials.

## The osteopathic audience

Not surprisingly, many within the osteopathic profession have chosen to publish in OMPC for the reasons described above, as demonstrated by the original research and other articles posted on our journal website (http://www.om-pc.com). *Osteopathic Medicine and Primary Care *will continue to work in conjunction with its 2010 Editorial Board (http://www.om-pc.com/edboard) to maintain OMPC's unparalleled standing within the osteopathic profession. However, it is important to remind our readers that OMPC was purposely established to be an autonomous, independent forum for osteopathic research and scholarly work. *Osteopathic Medicine and Primary Care *is not affiliated with or published by any national, state, or local osteopathic (or allopathic) professional associations, societies, or colleges. Nor is OMPC published by The Osteopathic Research Center or any other osteopathic (or allopathic) research institutes or centers. *Osteopathic Medicine and Primary Care *depends on the voluntary efforts of its editorial board members and peer reviewers to advance knowledge in the fields of osteopathic medicine and primary care. *Osteopathic Medicine and Primary Care *is not intended, per se, to fulfill an advocacy role for professionals within osteopathic medicine (or primary care).

## The primary care audience

The majority of osteopathic physicians are primary care physicians [1]; however, the majority of primary care physicians are not osteopathic physicians [2]. Hence, OMPC also seeks to serve the audience of allopathic physicians and other investigators and scholars in the field of primary care. In 2010, OMPC is especially interested in attracting additional manuscript submissions and publishing more research from authors representing this audience.

## The year of 2009 in review

The mean time from initial manuscript submission to publication of non-editorial articles in 2009 decreased to 118 days (our goal continues to be a mean 90-day submission-to-publication interval). Our rapid peer review during 2009 could not have been accomplished without dedicated individuals (Appendix), many of whom evaluated multiple manuscripts during the year. We also began posting book reviews on our journal website. To cover the cost of operations in 2009, OMPC continued to levy article processing charges (APCs) after a manuscript submission had been peer-reviewed and accepted for publication. However, authors at BioMed Central member institutions received a full waiver or partial discount of the APCs depending on their type of membership. Journal funds and discretionary waivers were also available in 2009 to help offset APCs.

We are pleased to announce that Clark and colleagues have been selected as recipients of the 2009 OMPC Article-of-the-Year Award for their publication entitled, "Muscle functional magnetic resonance imaging and acute low back pain: a pilot study to characterize lumbar muscle activity asymmetries and examine the effects of osteopathic manipulative treatment." The article was published in OMPC in August 2009 [3](http://www.om-pc.com/content/3/1/7). Hruby and Hoffman [4] and Licciardone and colleagues [5] were previously selected as the awardees for the years of 2007 and 2008, respectively.

## New initiatives during the year of 2010

As indicated above, OMPC is interested in publishing more articles in the realm of primary care medicine. We not only encourage submission of manuscripts that address the broad range of clinical aspects of primary care, but that also have relevance to health care delivery, public health, and evidence-based medicine. To facilitate publishing a greater number of articles relating to primary care, OMPC will substantially increase the monies available within its journal funds to provide waivers or discounts of APCs for authors who are not affiliated with BioMed Central member institutions and who do not otherwise have access to grant funds or local monies to support the cost of publication. All waivers, regardless of funding mechanism, must be requested at the time of initial manuscript submission. The two main criteria in determining whether a waiver is awarded are the quality of the submitted work and its congruence with our scope of interest. Additionally, we will post more items relevant to the fields of osteopathic medicine and primary care on the "Latest News" section of our journal website.

## Conclusions

*Osteopathic Medicine and Primary Care *strives to promote and advance research and scholarly work within the fields of osteopathic medicine and primary care. In so doing, OMPC welcomes submissions from clinicians within both the osteopathic and allopathic medical professions, and from other professionals having interests in primary care, including health care delivery, public health, and evidence-based medicine.

## Competing interests

The author declares that he has no competing interests.

## Appendix

*Osteopathic Medicine and Primary Care *peer reviewers during 2009: Roberto Cardarelli, Christine Choate, Michael Clearfield, Tyler Cymet, Gautam Desai, Robert Froud, Murray Goldstein, Stanley Grogg, Cheryl Hawk, Raymond Hruby, Cathleen Kearns, John Licciardone, Robert Moran, Harrison Ndetan, Paula Scariati, Michael Seffinger, Jill Slade, Clive Standen, Karen Steele, Melicien Tettambel, John Triano, Stephen Weis, Frank Willard.
